# Apatinib Mesylate in the treatment of advanced progressed lung adenocarcinoma patients with EGFR-TKI resistance —A Multicenter Randomized Trial

**DOI:** 10.1038/s41598-019-50350-6

**Published:** 2019-09-30

**Authors:** Ping Fang, Liqin Zhang, Xianru Zhang, Jiawen Yu, Jun Sun, Qi-an Jiang, Mingbao Zha, Anastasia P. Nesterova, Hongbao Cao

**Affiliations:** 1 Department of Respiratory, the People’s Hospital of Tongling, Tongling, Anhui province 244000 China; 2 Department of Respiratory, Yijishan Hospital of Wannan Medical College, No. 2 Zheshan West Road, Wuhu, Anhui Province 241000 China; 3 Department of Respiratory, Tongling Municipal Hospital, No. 2999 Changjiang West Road, Tongling, Anhui Province 244099 China; 4 Department of Respiratory, Anqing First People’s Hospital, No. 42 Xiaosu Road, Anqing, Anhui Province 246000 China; 5 Department of Respiratory, Xuancheng People’s Hospital, No. 15 Huancheng North Road, Xuancheng, Anhui Province 242000 China; 6 Department of Respiratory, Anqing Municipal Hospital, No.172 Renmin Road, Yingjiang District, Anqing, Anhui Province 246000 China; 7 Department of Respiratory, Wuhu City Hospital of Traditional Chinese Medicine, No. 240 Jiuhua Middle Road, Jinghu District, Wuhu, Anhui Province 2461002 China; 8 Department of Biology Solution, Elsevier, 1150 18th St NW, Washington, DC 20036 USA; 9 Department of Psychiatry, First Hospital/First Clinical Medical College of Shanxi Medical University, Taiyuan, Shanxi Province 030001 China

**Keywords:** Cellular signalling networks, Targeted therapies

## Abstract

Few pieces of evidence have been published on the use of Apatinib Mesylate (AM) against EGFR-TKI resistance in lung adenocarcinoma (LA) patients. Here, we investigate the clinical efficacy and safety of AM in the treatment of advanced progressed epidermal growth factor receptor tyrosine kinase inhibitors (EGFR-TKI) resistant LA patients. We conducted a double-blind, randomized controlled trial in 68 patients admitted to 18 hospitals of Anhui province in China. The efficacy and safety of AM treatment were evaluated in terms of progression-free survival (PFS), objective response rate (ORR), and disease control rate (DCR), as well as related adverse events (AE). A literature knowledge database analysis and a pathway model reconstruction were performed to decipher the relevant mechanism may be involved. Our results showed that, compared to the control group, AM presented improved efficacy in PFS (P = 0.033), ORR (P < 0.001), and DCR (P < 0.001). No significant difference was observed between case and control group in terms of AE, and no drug-related death occurred. Pathway analysis supports that Apatinib can be repurposed for the treatment of LA. Our results suggested that AM could be a potential option for advanced progressed LA patients to combat EGFR-TKI resistance.

## Introduction

Lung adenocarcinoma is one of the most common pathological patterns of lung cancer, which is often advanced by the time of diagnosis^1^. The mutation of epidermal growth factor receptor (EGFR), which is the first discovered and prototypical member of receptor tyrosine kinase family (RTK), is markedly prevalent (40–60%) in lung adenocarcinoma. Treatment with receptor-tyrosine kinase inhibitors (TKIs), particular EGFR targets, such as Gefitinib, Erlotinib, and Afatinib, have been explored to treat advanced lung adenocarcinoma during the past decades and show distinct treatment advantages over cytotoxic therapy at progression-free and overall survival^2^.

However, recent clinical practice demonstrates that such single mode of inhibition will inevitably develop into secondary resistance and treatment failure. Combined approaches using multiple kinase inhibition and vertical inhibition by combinations of small molecules and antibodies are testified to be more efficient to deal with the secondary EGFR–TKI resistance^3^. Bevacizumab, an anti-vascular endothelial growth factor (VEGF) therapy, is recommended to be the first-line therapy in lung adenocarcinoma in the National Comprehensive Cancer Network (NCCN) Clinical Practice Guidelines in Oncology. Apatinib Mesylate (AM; Hengrui Pharmaceutical Co. Ltd, Jiangsu, People’s Republic of China), a potent inhibitor of vascular endothelial growth factor receptor-2 (VEGFR-2 or kinase insert domain receptor, KDR), is developed and manufactured in China^4^, which can selectively inhibit VEGFR-mediated endothelial cell migration and proliferation, thus blocking new blood vessel formation in tumor tissue^5^. Previous researches also report that Apatinib Mesylate is effective in the treatment of various types of cancers with acceptable toxicities, such as gastric cancer, breast cancer, colorectal cancer, and esophageal cancer^6–9^.

To date, few reports have been published on the use of Apatinib Mesylate against EGFR-TKI resistance in lung adenocarcinoma patients^5^. Thus, this multicenter retrospective study is performed to investigate the safety and efficacy of Apatinib Mesylate in advanced lung adenocarcinoma patients after failure of prior EGFR-TKI treatment.

## Methods

### Trial design

In 18 medical units of Anhui Provinces, we randomized patients in a double-blind, comparing the clinical consequences of cases and controls in the treatment of advanced progressed EGFR-TKI resistant LA patients between Jun. 2016 and Sep. 2018. The study was approved by the Ethics Committee of the People’s Hospital of Tongling (IRB Number: TL201602). All methods were performed in accordance with the relevant guidelines and regulations.

### Eligibility criteria for participants

EGFR-TKI resistant patients were recruited according to the following inclusion criteria: (i) old than 18; (ii) primary lung adenocarcinoma diagnosed by pathology or cytology with at least one measurable tumor lesion with CT scan (long diameter ≥10 mm), lymph node lesion (short diameter ≥15 mm), and scan layer thickness ≤5 mm; (iii) underwent EGFR-TKIs treatment while the disease still progressed; (iv) Eastern Cooperative Oncology Group (ECOG) score: 0–2; (v) without apparent abnormalities in the routine blood tests and biochemical tests; (vi) expected survival ≥3 months; (vii) did not participate in other clinical research projects. Main exclusion criteria: (i) pregnant or lactating woman; (ii) patients with hypertension and cannot be reduced to the normal range by antihypertensive medication; (iii) bleeding tendency; (iv) symptomatic central nervous system metastasis.

### Interventions

Sixty-eight patients were randomly divided into two groups: (1) control group, treated with traditional chemotherapy drugs, such as pemetrexed (50 mg/m^2^, once every 21 days) and docetaxel (75 mg/m^2^, once every 21 days), alone or in combination with platinum (75 mg/m^2^, once every 21 days); (2) case group, treated with AM 500 mg/m^2^ per day for 21 consecutive days) or AM combined with traditional chemotherapy drugs. The patients in both groups were treated for 21 days as a treatment cycle.

The patients’ disease progression status was followed up until September 30, 2018, or death. Informed consents were signed by all patients, and this study was approved by the ethics committee of each hospital involved. Patients’ demographic and baseline clinical characteristics including age, sex, ECOG score, line of treatment, metastatic status, primary surgery history, number of metastasis, EGFR mutation, and radiotherapy history were collected and analyzed.

### Outcomes of efficacy and safety evaluation

The short-term efficacy was evaluated every 2 cycles (42 days) of the treatment as in terms of complete remission (CR), partial remission (PR), disease stability (SD), disease progression (PD), progression-free survival (PFS), disease control rate (DCR) and objective response rate (ORR), as well as related adverse events (AE) evaluated by the National Cancer Institute Common Toxicity Criteria version 4.0 (NCI-CTC 4.0).

### Patient recruitment and randomization

From Jun. 2016 to Sep. 2018, we recruited 90 patients who were diagnosed with lung adenocarcinoma. Of these, 21 were excluded from the study because of not meeting one or more filtering criteria. The remaining cohort consisted of 68 patients met the criteria of our screening.

Eligible patients were randomly assigned to case and control groups. A unique study ID was randomly signed to each of the participants. Randomized patients received treatment during the study period, according to the intervention they were allocated. Study investigators, research coordinators, attending care teams, and the patients were blinded to treatment allocation.

### Statistical methods

Baseline characteristics of patients in the two treatment groups were reported using frequency distributions. Kaplan-Meier method was used to estimate progression-free survival (PFS), time to progression, or overall survival (OS), which showed time following treatment on the horizontal axis, and the probability of surviving on the vertical axis. The log-rank test was used to compare the difference of survival rate between case and control groups with a χ^2^ test of the null hypothesis, which first calculated the sum of (O-E)^2^/E for each group, where O and E were the totals of the observed and expected events, then the *p*-value was got from the χ^2^ distribution table. The *p*-value ≤ 0.05 was considered as statistically significant. The data were processed using SPSS17.0 statistical software (SPSS Inc., Chicago, IL, USA).

### Sample size and power calculation

The null hypothesis of this study was that the survival rate for case and control groups are equal (p0 = 0.5), and we set a reject p-value as 0.05. For using χ^2^ test to test this hypothesis, we used Matlab statistical analysis toolbox for sample size and power calculation for hypothesis test (Matlab 2017a; function ‘sampsizepwr()’). Results showed that a sample size of 7 is required to have the power to reject the null hypothesis. Therefore, the sample size in this study was big enough to provide statistical power for the analysis (case/control: 39/29).

### Influential factors analysis

A multiple linear regression analysis has been conducted to study potential influential factors for both the adverse effects and treatment efficacy, including sex, age, medical history, radiotherapy history, and prior treatment. Results were reported with significant factor selection criterion set as p < 0.05.

### Pathway analysis

Analysis of Apatinib interactions with molecules was based on a literature-based knowledge database (Pathway Studio, www.pathwaystudio.com), which covers more than 50 million records from the published papers and updates weekly^10^. Images and interactive pathway models were built in Pathway Studio to explore the molecular mechanisms of how Apatinib can inhibit tumor growth in LA.

### Trial registration

The trial was registered at the US National Institutes of Health (ClinicalTrials.gov, #NCT03083041) on March 17, 2017.

### Data access and responsibility

The principal investigator, Ping Fang, had full access to all of the data in the study and takes responsibility for the integrity of the data and the accuracy of the data analysis.

### Ethical approval and informed consent

This study was approved by the Ethics Committee of the People’s Hospital of Tongling (IRB Number: TL201602). We obtained written informed consent from all participants after explaining them the study protocol.

## Results

### Clinical characteristics

**Figure 1:**
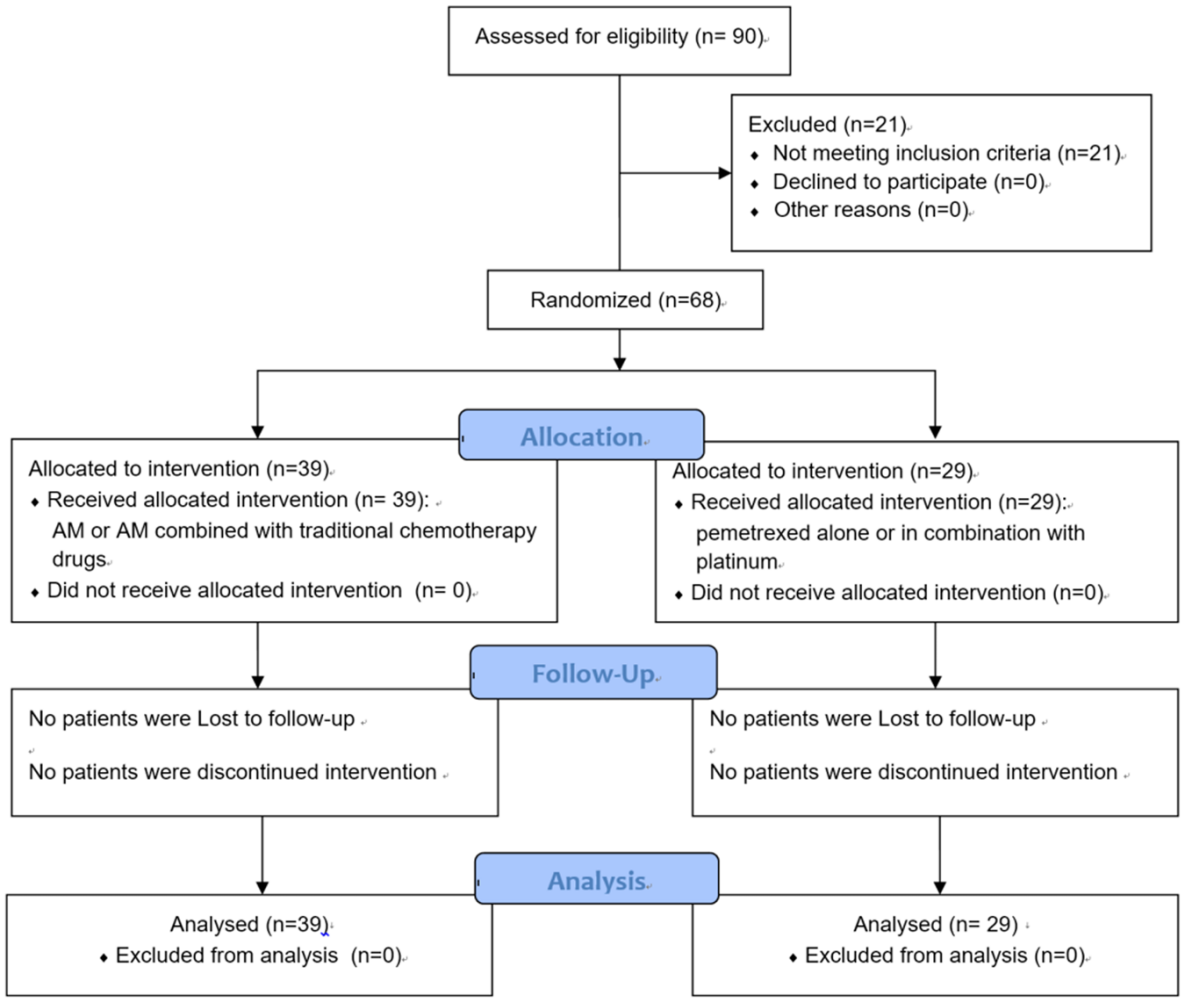
Workflow Diagram to evaluate the treatment effect of Apatinib Mesylate on advanced progressed lung adenocarcinoma patients with EGFR-TKI resistance.

A total of 90 patients were screened for eligibility, and 68 met eligibility criteria and were randomized between Jun. 2016 and Sep. 2018 (Fig. 1). The 38 patients and 29 controls were randomly signed into case or control groups, respectively. All randomized patients completed the trial. The average ages for both groups were 60.2 and 62.8, and 59.0% and 58.6% of the patients were female. Most patients presented good ECOG score. There were 48 out of 68 patients presented positive EGFR exon mutation: 44.1% in 19 exons and 26.5% in 21 exons. Baseline demographics and characteristics were summarized and shown in Table 1.

**Table 1:** The characteristics of 68 patients in case and control groups.

Characteristics		Case (39) N (%)	Control (29) N (%)
Age	Mean ± SD	60.2 ± 11.8	62.8 ± 11.6
Gender	Male	16 (41.0)	12 (41.4)
	Female	23 (59.0)	17(58.6)
ECOG Score	0	2 (5.2)	0
	1	16 (41.0)	6 (20.7)
	2	21 (51.8)	23 (79.3)
Treatment line	≤4	26 (66.7)	29 (100)
	>4	13 (33.3)	0
Brain metastasis	Yes	13 (33.3)	9 (31.0)
	No	26 (66.7)	20 (68.9)
Primary surgery	Yes	5 (12.8)	6 (20.7)
	No	34 (87.2)	23 (79.3)
Number of metastatic sites	≤2	27 (69.2)	27 (93.1)
	>2	12 (30.8)	2 (6.9)
EGFR mutation	19 exon	12 (30.8)	18 (62.1)
	21 exon	7 (17.9)	11 (37.9)
	Unclear	20 (50.3)	0
Previous radiotherapy treatment	Yes	20 (51.3)	4 (13.8)
	No	19 (48.7)	25 (86.2)

### Short-term efficacy

**Figure 2:**
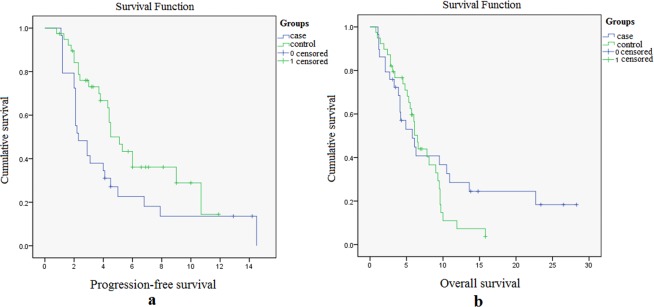
The survival charts. (**a**) PFS of the case and control groups: 5.1 months vs. 2.3 months, p = 0.033; (**b**) the OS of the case and control groups: 6.5 months vs. 5.8 months, p > 0.05.

The follow-up lasted until September 2018, and all the 68 patients could be evaluated for efficacy. The values of CR in case and control groups were 0, 11 vs. 1 in PR, 19 vs. 5 in SD, and 9 vs. 23 in PD. The total DCR and ORR in the case group were significantly higher than the control group (*p* < 0.001, Table 2). The results indicated that AM had a better impact on lung adenocarcinoma patients who underwent EGFR-TKIs treatment while the disease still progressed. Kaplan-Meier method was performed to reveal the survival function of PFS and OS in case and control groups. Further, Log-rank test was used to compare the survival distributions (For details of the statistical analysis, please refer to Supplementary Material 1). Compared with 2.3 months in the control group, the median PFS in the case group was prolonged to 5.1 months (95% CI: 3.9–6.3, *P*-value = 0.033, Fig. 2a). Besides the cumulative anti-tumor effects, the reasons for AM prolongs PFS may be associated with its effects of inhibiting the VEGFR-2 action, reversing the EGFR-TKIs drug resistance and enhancing the body’s sensitivity. However, the median OS did not show any significant differences between the two groups (6.5 months vs. 5.8 months, *p* > 0.05, Fig. 2b). All of these indicated that AM could improve the short-term effect of EGFR-TKI resistant adenocarcinoma patients.

**Table 2:** The short-term efficacy comparison between case and control groups.

	Case Group	Control Group	P-Value
CR	0	0	
PR	11	1	
SD	19	5	
PD	9	23	
DCR(CR + PR + SD)	30 (76.9%)	6 (20.7%)	p < 0.001
ORR (CR + PR)	11 (28.2%)	1 (3.4%)	p < 0.001

### Safety evaluation

Besides the short-term efficacy, the safety evaluation based on the incidence of adverse events has been considered, with results presented in Table 3. There were no patient-death during the treatment period. The overall incidence of adverse events was comparable (89.7% vs. 96.6%, *p* > 0.05) and the rate of serious adverse events (grade 3 or 4) in case and control were 33.3% (13/39) and 13.8% (4/29) (*p* < 0.05). Furthermore, the main adverse events of case group were mostly mild adverse reactions and can be managed. For the top three serious adverse events in the case group were fatigue, hypertension, hand-foot syndrome, and proteinuria, while the main adverse event in the control group was the gastrointestinal reaction. To note, there were no significant differences between case and control group in terms of the adverse event.

**Table 3:** The comparison of AE between case and control groups.

AE Grade	Case (n = 39)			Control (n = 29)		
	1–2	3–4	n (%)	1–2	3–4	n (%)
Hypertension	11	4	15 (38.5)	2	0	2 (6.9)
Hand-foot syndrome	15	3	18 (46.2)	6	1	7 (24.1)
Proteinuria	4	3	7 (18)	0	0	0
Fatigue	5	5	10 (25.6)	0	3	3 (10.3)
Lung infection	1	0	1 (2.6)	1	0	1 (3.4)
Bone marrow suppression	1	0	1 (2.6)	1	0	1 (3.4)
Oral ulcer	2	0	2 (5.1)	1	0	1 (3.4)
Cough	1	0	1 (2.6)	0	0	0
Bilirubin increase	1	0	1 (2.6)	1	0	1 (3.4)
Thrombocytopenia	1	0	1 (2.6)	1	0	1 (3.4)
Bleeding	4	2	6 (15.4)	1	0	1 (3.4)
Gastrointestinal reaction	1	0	1 (2.6)	27	0	27 (93.1)
Anemia	2	0	2 (5.1)	1	0	1 (3.4)
**Serious AE rate**	13 (33.3)			4 (13.8)		
**Total AE rate**	35 (89.7)			28 (96.6)		

### Influential factors for adverse effects

MLR results showed that age is positively related to the overall adverse effects when using Apatinib treatment (p < 0.01), while age, medical history, radiotherapy history, and prior treatment history were not (p > 0.5), as shown in Table 4. No significant factor was observed for the treatment effect (p > 0.13).

**Table 4:** Multiple Linear Regression analysis results for influential factors of the adverse effect of Apatinib treatment.

	MLR parameters	Sex	Age	Medical History	Radiotherapy History	Prior Treatment
Adverse Effects	Beta	−0.23	0.02	−0.61	−0.45	0.00
	Low Limit	−0.64	0.01	−1.02	−0.85	0.00
	Up Limit	0.17	0.04	−0.20	−0.05	0.00
	p-value	0.90	<0.01	1.00	1.00	0.50
Treatment efficacy	Beta	−0.03	0.011	−0.25	0.001	0.00
	Low Limit	−0.58	−0.01	−0.80	−0.54	0.00
	Up Limit	0.52	0.04	0.31	0.55	0.00
	p-value	0.54	0.13	0.84	0.49	0.5

### Pathway analysis

**Figure 3:**
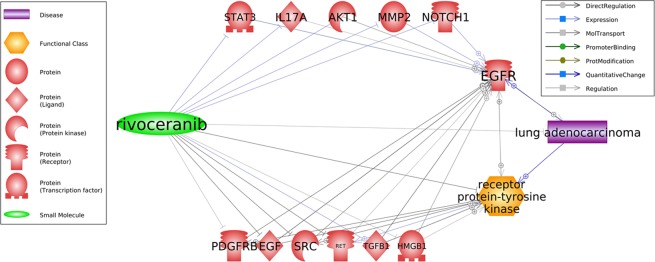
Pathways revealing potential mechanisms how Apatinib (rivoceranib) can treat EGFR-TKI resistant lung adenocarcinoma.

As shown in Fig. 3, Apatinib (rivoceranib) has been reported to present a treatment effect on LA. This pathway has been generated by using Pathway Studio (www.pathwaystudio.com). Moreover, Apatinib has been shown to inhibit multiple common promoters of EGFR/TI, including PDGFR, BEGF, SRC, RET, TGFB1, and HMGB1. These could be the possible mechanisms explaining how Apatinib could treat (EGFR-TKI) resistant LA. Moreover, we explored additional literature-based functional pathways to understand the treatment effect of Apatinib on lung adenocarcinoma (see Supplementary Material 2).

## Discussion

Angiogenesis plays a significant role in tumor development, while the VEGF signaling is an important regulator of angiogenesis^11,12^, therefore, targeting angiogenesis by inhibition of VEGF and or VEGFR is a potential treatment option for many cancers. Pathology of VEGF signaling in cancers is characterized by hyperproduction of VEGF ligand family (VEGF-A, VEGF-B, VEGF-C, VEGF-D) by tumor and tumor-infiltrating cells. Simultaneously, blood vessel endothelial cells are overstimulated to express more VEGF receptors (VEGFR1, VEGFR2, and VEGFR3). As a result, VEGF and VEGFRs are highly expressed in various tumor vascular endothelium and lymphatic vessels^13^ resulting in new blood vessel formation and additional oxygen supply of the growing tumor. Tumor-associated hypoxia and increased expression of oxygen-regulated HIF transcription factors are the important inducers of VEGFA and VEGFR1 overexpression. Inflammatory cytokines, growth factors, hormones, and mutations of the RAS proto-oncogenes also lead to hyperexpression of VEGFs in cancer^14,15^.

As a novel oral TKI targeting VEGFR2, Apatinib Mesylate can significantly inhibit the angiogenesis of neoplasms and has been demonstrated to be tolerance, safety, and efficacy in the clinic^16–18^. Apatinib binds to intracellular VEGFR2 domains inhibiting proliferation, migration, and tube formation of vascular endothelial cells^4^. Although VEGFR2 is known to be expressed mostly in epithelial cells, its overexpression and the tumorous effect was shown in several cancer types including gastric and endometrial cancer^14–19^. Apatinib treatment inhibits VEGFR2 - mediated proliferation, migration, or invasion of liver cancer cells^20^ or cholangiocarcinoma cells^21^. In addition, it induces VEGFR2 - related apoptosis and autophagy in cell models of osteosarcoma^22^ and intrahepatic cholangiocarcinoma^23^.

VEGFR2 binds to VEGF-E, VEGF-C, and VEGF-D and to lower molecular weight forms of VEGF-A (110–165 amino acid residues). Activated VGFR2 (KDR, FKL1, Fetal liver kinase-1/Kinase Domain-containing Receptor) activates several prominent intracellular cascades which control protein translation, cell cycle, and cellular cytoskeleton^24,25^. In gastric cancer cells, VEGFR2 transfers signals through the activation of PLCG1 and MAPK1/3 pathways^26^.

Apatinib could efficiently inhibit the VEGFR2/RAF/MAPK1/3 signaling, which controls general cell proliferation in several cancer cells. In intrahepatic cholangiocarcinoma Apatinib inhibits VEGFR2 mediated PI3K/AKT/mTOR signaling pathway which is a central regulator of cellular translation^23,27–29^. In liver cancer and osteosarcoma, Apatinib inhibits VEGFR2/STAT3/BCL2 pathway leading to a tumor cell, apoptosis, and autophagy^22^. VEGF receptors modulate the activity of each other but may have different roles in cancer progression. For example, in cancer ICC cells, only VEGFR2 (not VEGFR1) blocks the apoptosis through PI3K anti-apoptotic signaling pathway. In contrast, treatment only with anti-VEGFR1 but not with anti-VEGFR2 antibody prevented the formation of pre-metastatic sites in animal model^14^.

Effects of Apatinib Mesylate could not be VEGFR2 - depended, since it also mildly inhibits KIT proto-oncogene receptor tyrosine kinase, SRC proto-oncogene, nonreceptor tyrosine kinase, and RET proto-oncogene receptor tyrosine kinase^4^. Currently, Apatinib Mesylate is testified in chemotherapy-refractory advanced metastatic gastric cancer (phase II/III clinical trials) in China^18^, which is the only effective drug for patients with terminal gastric cancer who have no other chemotherapeutic options^26^. Apatinib Mesylate is also promising for advanced lung cancer, metastatic breast cancer, and advanced hepatocellular carcinoma (the phase I/II/III clinical trial)^30^.

A recent study also indicates that Apatinib Mesylate might be an option for post-first-line treatment of EGFR wild-type advanced lung adenocarcinoma^31^. Moreover, there are three ongoing clinical trials conducted in recent years to test the effect of Apatinib in advanced lung adenocarcinoma [NCTID: NCT02493582; NCT02691871; NCT03376737]. Advanced lung adenocarcinoma is characterized not VGFR2-hyperactivated but altered EGFR signaling pathway. Though, VEGFR2 shares with EGFR key intracellular partners such as SHC1, protein tyrosine kinase 2, and phospholipase C. EGFR-mutant lung adenocarcinoma patients who acquired EGFR-TKI treatment resistance may benefit from blocking VEGFR2 related angiogenesis and tumor growth. It is worth noting that Apatinib also reverses the multidrug resistance (MDR) condition in several cancer cell lines by the inhibition of ATP Binding Cassette Subfamily B Member 1 (ABCB1) and ATP-binding cassette superfamily G member 2 (ABCG2). ABCB1 and ABCG2 when overexpressed, transport chemotherapeutic drugs out of cancer cell. The mechanism of ABCB1 and ABCG2 inhibition by Apatinib is still unclear. Apatinib may be a direct substrate of both ABCB1 and ABCG233. In non-small cell lung cancer Apatinib reverses the drug resistance to gefitinib because of different mechanisms: synergistic effect on VEGFR2 and EGFR signaling pathways; or due to Warburg effect when a high rate of glycolysis exhausts the tumor growth^32^. B-cell lymphoma 2 (Bcl-2), which is a downstream target of VGFR2 pathway, may also participate in lung adenocarcinoma MDR and be modified by Apatinib treatment^33^. So, the precise mechanism involved in the sensitivity of Apatinib Mesylate mediated TKI resistance need further investigation.

EGFR-TKIs-challenged therapy for EGFR-mutant lung adenocarcinoma patients who acquired treatment resistance shows only moderate efficacy, and there were few pieces of research focused on the effect of Apatinib on EGFR-TKI resistance patients. In light of the high interrelation between EGFR and VEGF/VEGFR pathways and potential angiogenesis function, the treatment effect of Apatinib Mesylate is evaluated in EGFR-TKI-resistant patients in this research. The patient benefited another 5.1-month PFS after confronting EGFR-TKI resistance. Thus, these findings demonstrate the treatment advantage of Apatinib Mesylate alone or combined with chemotherapeutic agents, and all of these suggest that Apatinib Mesylate is a viable choice for the treatment of advanced progressed lung adenocarcinoma patients with EGFR-TKI resistance.

However, due to the limited sample size, the comparison between the effects of Apatinib Mesylate alone and Apatinib Mesylate combined with chemotherapy is not performed. Our study showed that Apatinib could have the adverse effect of causing hypertension (38.50%), hand-foot syndrome (46.20%), and proteinuria (18.00%) in the treatment of advanced progressed LA with EGFR-TKI resistance. However, the ratios of patients affected by these adverse effects are different from a previous 34-patients research^34^ (hypertension: 35.30%, hand-foot syndrome 23.53%, and proteinuria: 14.71%). Moreover, another investigation (40 patients)^35^ found that Apatinib can lead to 17.5% hypertension, 30.0% hand-foot-skin reaction, and 27.5% proteinuria in pretreated advanced non-squamous non-small-cell lung cancer. It requires further study with a bigger-sample-sized and multi-population study to test whether such discrepancy is due to pretreatment, EGFR-TKI resistance, or patient characteristic.

Pathway analysis using Pathway Studio (www.pathwaystudio.com) supported our results and identified multiple pathways through which AM could inhibit the promoters of EGFR/TKI. This partially explains the mechanism of how AM could be used for EGFR-TKI resistant LA patients.

In conclusion, our study indicates that Apatinib Mesylate may have good therapeutic efficacy in treating patients with advanced progressed lung adenocarcinoma with EGFR-TKI resistance, and the adverse events can likely be controlled.

## Supplementary information

Consort 2010 Checklist

Consort 2010 Flow Diagram

Protocol

Supplementary Materials 1

Supplementary Materials 2
