# Supplementary material for: Apatinib Mesylate in the treatment of advanced progressed lung adenocarcinoma patients with EGFR-TKI resistance —A Multicenter Randomized Trial
Source: Sci Rep. 2019 Sep 30;9:14013. doi: 10.1038/s41598-019-50350-6 (PMC6768876; doi:10.1038/s41598-019-50350-6)
Supplement: Supplementary file 2 — Consort 2010 Flow Diagram [file 41598_2019_50350_MOESM2_ESM.doc]

**
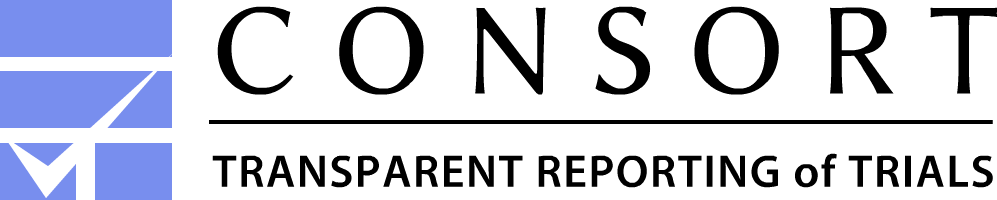
**

**CONSORT 2010 Flow Diagram**

**Allocation**

**Analysis**

**Follow-Up**

**Enrollment**

Assessed for eligibility (n= 90)

Excluded (n=21)

♦  Not meeting inclusion criteria (n=21)

♦  Declined to participate (n=0)

♦  Other reasons (n=0)

Analysed (n=39)
♦ Excluded from analysis (n=0)

No patients were Lost to follow-up

No patients were discontinued intervention

Allocated to intervention (n=39)

♦ Received allocated intervention (n= 39):

AM or AM combined with traditional chemotherapy drugs

♦ Did not receive allocated intervention (n= 0)

No patients were Lost to follow-up

No patients were discontinued intervention

Allocated to intervention (n=29)

♦ Received allocated intervention (n=29):

pemetrexed alone or in combination with platinum

♦ Did not receive allocated intervention (n=0)

Analysed (n= 29)
♦ Excluded from analysis (n=0)

Randomized (n=68)
