# Supplementary material for: Apatinib Mesylate in the treatment of advanced progressed lung adenocarcinoma patients with EGFR-TKI resistance —A Multicenter Randomized Trial
Source: Sci Rep. 2019 Sep 30;9:14013. doi: 10.1038/s41598-019-50350-6 (PMC6768876; doi:10.1038/s41598-019-50350-6)
Supplement: Supplementary file 3 — Protocol [file 41598_2019_50350_MOESM3_ESM.docx]

**Clinical study of Apatinib Mesylate in the treatment of lung adenocarcinoma with EGFR-TKIs-resistant**

Edition：1.0

Original Protocol: July 25 2016

**Sponsor**: Jiangsu Hengrui Pharmaceutical Co., Ltd

Address: 7 Kunlun Mountain Road, Lianyungang Economic and Technological Development Zone, Lianyungang, Jiangsu Province, 222047, China.

**Principle Investigator**: Ping Fang

Research Site: The People’s Hospital of Tongling, Anhui Province, China

Address: 468 Bijiashan Rd, Tong Guan Shan Qu, Tongling Shi, Anhui Sheng, China, 244004

Phone: +86 562 282 5534

**CONFIDENTIALITY STATEMENT**

The information in this document contains trade secrets and commercial information that are privileged or confidential and may not be disclosed unless such disclosure is required by applicable law or regulations. In any event, persons to whom the information is disclosed must be informed that the information is privileged or confidential and may not be further disclosed by them. These restrictions on disclosure will apply equally to all future information supplied to you which is indicated as privileged or confidential.

**Project summary**

| Subjects | Lung adenocarcinoma with EGFR-TKIs-resistant |
| --- | --- |
| Objects | The objective of this study is to observe and evaluate the efficacy and safety of apatinib mesylate in the treatment of lung adenocarcinoma with EGFR-TKIs-resistant |
| Sample Size | At least 50 qualified subjects |
| Study Duration | August 2016- August 2018 |
| Patient screening criteria | **Inclusion criteria：**   - 1. Age: ≥ 18 years old, male or female;   2. Patients with primary lung adenocarcinoma confirmed by pathology or cytology have at least one measurable lesion (CT scan with a long diameter of ≥10 mm, CT scan with a short diameter of ≥15 mm, and a scan layer thickness of no more than 5 mm);   3. EGFR mutation is positive;   4. Resistance to treatment with EGFR-TKIs (including but not limited to Iressa and/or AZD9291);   5. ECOG score: 0-2 points;   6. Estimated survival time ≥ 3 months;   7. The main organs function normally   **Exclusion criteria：**   - 1. Pregnant or lactating women;   2. Patients with hypertension and antihypertensive medication cannot fall to the normal range (systolic blood pressure >140 mmHg, diastolic blood pressure >90 mmHg) with grade I or higher myocardial ischemia or myocardial infarction, arrhythmia and grade II heart Incomplete function;   3. abnormal blood coagulation (INR > 1.5 or prothrombin time (PT) > ULN + 4 seconds or APTT > 1.5 ULN), with bleeding tendency or receiving thrombolytic or anticoagulant therapy;   4. Have clear gastrointestinal bleeding concerns (such as local active ulcer lesions, fecal occult blood + +), a history of gastrointestinal bleeding within 6 months;   5. Symptomatic central nervous system metastasis;   6. Pulmonary hemorrhage ≥ CTCAE level 2 occurred within 4 weeks prior to the first use of the study drug; bleeding occurred at other sites of grade ≥ CTCAE 3 within 4 weeks prior to the first use of the study drug. |
| Study Design | Subjects were randomly divided into two groups: 1) control group, treated with traditional chemotherapy drugs, such as pemetrexed (50 mg/m^2^, once every 21 days) and docetaxel (75 mg/m^2^, once every 21 days), alone or in combination with platinum (75 mg/m^2^, once every 21 days); 2) case group, treated with Apapitatin Mesylate (AM) 500 mg/m^2^ per day for 21 consecutive days) or AM combined with traditional chemotherapy drugs. The patients in both groups were treated for 21 days as a treatment cycle.  The patients were contiuously treated by AM for 21 days until PD, intolerable toxicity or patient request to stop; Daily oral dexamethasone 8 mg, 12 h/time before docetaxel, for 3 days. Docetaxel 60~75mg/m2, intravenous infusion for 1 hour, repeated every 3 weeks; or weekly therapy, docetaxel 35～40mg/m2, intravenous infusion for 1 hour, once a week, continuous use 6 weeks, stop for 2 weeks.  When apapitatin mesylate is combined with TKIs, dose adjustment can be made according to the patient's condition and systemic condition. |
| Main efficacy index | Progression-free survival (PFS) |
| Secondary efficacy index | Overall survival (OS), disease control rate (DCR), objective response rate (ORR)，Quality of Life (QoL). |
| Main safety indicator | Abnormalities in clinical symptoms and vital signs, abnormalities in laboratory tests, clinical manifestations, severity, time, duration, treatment and prognosis were recorded, and the correlation between them and the test drugs was determined. The safety of the drug was evaluated using the NCI-CTC AE version 4.0 standard. |

#### Background

#### Lung cancer has become the leading cause of cancer deaths worldwide, both male and female. According to the statistics of the 2013 China Cancer Registration Annual Report, the incidence of lung cancer in China is 51/100,000 and the mortality rate is 43/100,000. Whether it is tumor morbidity or mortality, lung cancer takes the first place, seriously threatening the health of the people.

#### The cause of lung cancer is still not fully understood. A large amount of data indicates that long-term large-scale smoking has a very close relationship with the occurrence of lung cancer. Long-term smokers are 10 to 20 times more likely to develop lung cancer than non-smokers and the younger the age at which they start smoking, the higher the risk of developing lung cancer. At the same time, smoking will also have an adverse effect on the health of the surrounding population, increasing the prevalence of lung cancer in passive smokers. In addition to smoking, exhaust gas, haze and industrial pollution are also important factors leading to an increase in the incidence of lung cancer. There is evidence that the haze weather can increase the risk of lung cancer death by 50% compared to the absence of PM2.5. This is also the lung cancer of urban residents. The incidence of morbidity is higher than that of rural areas [1].

#### Non-small cell lung cancer (NSCLC) includes squamous cell carcinoma (squamous cell carcinoma), adenocarcinoma, and large cell carcinoma. Compared with small cell carcinoma, its cancer cells grow slowly and have a relatively slow diffusion and metastasis. As a common histological type of lung cancer, lung adenocarcinoma accounts for about 50% of lung cancer and belongs to non-small cell lung cancer (NSCLC), which is one of the most common lung cancers. Non-small cell lung cancer disease is difficult to detect early, most of them are inoperable middle and advanced patients, and nearly 3/4 of these patients will receive chemotherapy [2].

#### According to the NCCN and Chinese lung cancer treatment guidelines, the current domestic NSCLC is mainly treated by drugs. Platinum-based dual-drug treatment has become the standard first-line treatment. Targeted drug therapy can be selected for patients with confirmed epidermal growth factor (EGFR) mutations. The second-line treatment option is docetaxel, pemetrexed or the targeted drug EGFR-TKI monotherapy. Third-line drugs can be treated with EGFR-TKI or clinical trials [3,4].

#### The NSCLC with EGFR kinase domain mutations shows that the histological characteristics of adenocarcinoma are highly sensitive to TKI and have a better prognosis than wild type. EGFR small molecule inhibitors erlotinib, gefitinib, afatinib, etc., can significantly delay disease progression in EGFR mutants [5-7]. Unfortunately, the median effective time of TKI treatment is only 5 to 9 months, and patients will eventually develop TKI resistance, relapse or progression [8]. How to overcome the TKI resistance strategy has become an urgent problem to be completed.

#### The combination of anti-angiogenic macromolecular antibodies (Ramucirumab) and small molecule inhibitors (Nintedanib) with docetaxel has achieved good clinical results in advanced lung cancer, significantly prolonging the survival of patients with NSCLC [9,10]. These results suggest that anti-tumor angiogenesis drugs provide a basis for anti-tumor angiogenesis drugs in the treatment of third- and fourth-line NSCLC.

#### In order to find high-efficiency, low-toxic anti-tumor angiogenesis drugs, Jiangsu Hengrui Pharmaceutical Co., Ltd. developed a highly potent VEGFR2 tyrosine kinase inhibitor, apatinib. The drug mainly inhibits VEGFR2 and exerts anti-angiogenic effect to treat malignant tumors. Both in vitro and in vivo tests show that apatinib has good tumor growth inhibitory activity against lung cancer. This study aims to further confirm the efficacy and safety of apatinib mesylate in the treatment of EGFR-TKIs-resistant lung adenocarcinoma.

#### References:

1. World Cancer Report 2014.WHO.
2. 2013 China Cancer Registration Annual Report.
3. Primary lung cancer diagnosis and treatment specification (2011 edition).
4. 2014 Non-small cell lung cancer guide.
5. Mok TS，Wu LL，Thongprasert S，et al．Gefitinib or carboplatin-paclitaxel in pulmonary adenocarcinoma．N Engl J Med，2009，36(1) :947-957．
6. Douillard JY，Shepherd FA，Hirsh V，et al．Molecular predictors of outcome with gefitinib and docetaxel in previously treated non-small-cell-lung-cancer: date from the randomized phase III IN-TEST trial．J Clin Oncol，2010，28( 5) : 744-752．
7. Cappuzzo F，Ciuleanu T，Stelmakh L，et al．Erlotinib as mainte-nance treatment in advanced non-small-cell-lung-cancer: a multi-centre，randomised，placebo- controlled phase 3 study．Lancet Oncol，2010，11( 6) :521-529．
8. Balak MN，Gong Y，Ｒiely GJ，et al．Novel D761Y and common secondery T790M mutation lung adecarcinomar with acquired re-sistancr to kinase inhibitions．Clin Cancer Ｒes，2007，13( 11) : 3431-3432．
9. M. Reck, R. Kaiser, A. Mellemgaard, J.-Y. Douillard, S. Orlov, M. Krzakowski, J. von Pawel, M. Gottfried, I. Bondarenko, M. Liao, Docetaxel plus nintedanib versus docetaxel plus placebo in patients with previously treated non-small-cell lung cancer (LUME-Lung 1): a phase 3, double-blind, randomised controlled trial, The lancet oncology, 15 (2014) 143-155.
10. E.B. Garon, T.-E. Ciuleanu, O. Arrieta, K. Prabhash, K.N. Syrigos, T. Goksel, K. Park, V. Gorbunova, R.D. Kowalyszyn, J. Pikiel, Ramucirumab plus docetaxel versus placebo plus docetaxel for second-line treatment of stage IV non-small-cell lungcancer after disease progression on platinum-based therapy (REVEL): a multicenter,double-blind, randomised phase 3 trial, The Lancet, (2014).

#### Study goals and objectives

- 1. **Research purposes**

To observe and evaluate the efficacy and safety of apapitatin mesylate combined with docetaxel in the treatment of EGFR-TKIs-resistant lung adenocarcinoma.

- 1. **Main study endpoint**

Progression free survival (PFS) is the date from the random date to the first occurrence of disease progression or death from any cause, whichever occurs first.

Definition of evaluable population: All subjects who met the following criteria, ie, received at least 2 treatment cycles (6 weeks) with apatinib and received 1 tumor evaluation after 2 treatment cycles (6 weeks) If the effect is CR, PR or SD, the subject should be reviewed 3 weeks after the first evaluation.

If the subject did not develop disease during the trial, the PFS was defined as the last date until the subject confirmed the last progression free survival. Subjects who discontinued the test (without follow-up imaging) for reasons other than progression of the disease and subjects who received the post-test treatment will be censored as the time to suspend the trial or the time to start the post-test treatment. When the subject is not censored at the time of the discontinuation of the trial or the time of initiation of the post-experimental treatment, the pre-planned sensitivity statistical analysis will further confirm the PFS based only on the time of occurrence of the imaging-confirmed progression. New tumors are not considered to be disease progression events and are not censored as data.

If imaging examinations and evaluations show disease progression, the disease progression date is not the first imaging time to show disease progression, but rather the time of imaging examination to confirm the progression of the disease. If the disease progression is diagnosed by other clinical means, the date of diagnosis will be the date of disease progression.

- 1. **Secondary study endpoint**

***Total survival (OS)*** is the date from a random date to any cause of death. For subjects who survived at the last follow-up, their OS was censored as data at the last follow-up. In the subjects who were lost to follow-up, the OS was counted as data censored by the last confirmed survival time before the loss of follow-up. The data censored OS is defined as the time from random grouping to censoring.

***Objective response rate (ORR)*** refers to the proportion of patients whose tumors have shrunk to a certain level and maintained for a certain period of time, including cases of CR and PR. Objective tumor remission assessment criteria (RECIST 1.1 criteria) were used to assess objective tumor remission. Subjects must be accompanied by measurable tumor lesions at baseline, and the efficacy criteria were divided into complete response (CR), partial response (PR), stable (SD), and progression (PD) according to RECIST 1.1 criteria.

***Disease Control Rate (DCR)*** refers to the percentage of confirmed complete remission, partial remission, and stable disease (≥ 8 weeks) in the number of patients with evaluable efficacy.

***Drug safety:*** Observed any adverse events that occurred in all subjects during the clinical study, including abnormalities in clinical symptoms and vital signs, abnormalities in laboratory tests, and recorded clinical manifestations, severity, time, duration, treatment, and prognosis. And determine its correlation with the test drug. The safety of the drug was evaluated using the NCI-CTC AE version 4.0 standard.

1. **Study Design**

This is a randomized multicenter study evaluating the safety and effectiveness of Apatinib Mesylate in the treatment of advanced progressed epidermal growth factor receptor-tyrosine kinase inhibitors (EGFR-TKI) resistant lung adenocarcinoma (LA) patients.

- 1. **Study Population**

The Investigator is expected to invite all subjects expected to meet the study entry criteria to participate in the study. Enrollment will be monitored by the Sponsor and the Investigators will be notified by the Sponsor once those targets have been met.

Eligible subjects will be enrolled and randomized divided into two groups: case and control groups. The patients in both groups were treated for 21 days as a treatment cycle.

- 1. **Subject Selection**

***3.2.1 Inclusion criteria：***

1. Age: ≥ 18 years old, male or female;
2. Patients with primary lung adenocarcinoma confirmed by pathology or cytology have at least one measurable lesion (CT scan with a long diameter of ≥10 mm, CT scan with a short diameter of ≥15 mm, and a scan layer thickness of no more than 5 mm);
3. EGFR mutation is positive;
4. Resistance to treatment with EGFR-TKIs (including but not limited to Iressa and/or AZD9291);
5. ECOG score: 0-2 points;
6. Estimated survival time ≥ 3 months;
7. The main organs function normally

***3.2.2 Exclusion criteria：***

1. Pregnant or lactating women;
2. Patients with hypertension and antihypertensive medication can not fall to the normal range (systolic blood pressure >140 mmHg, diastolic blood pressure >90 mmHg) with grade I or higher myocardial ischemia or myocardial infarction, arrhythmia and grade II heart Incomplete function;
3. abnormal blood coagulation (INR > 1.5 or prothrombin time (PT) > ULN + 4 seconds or APTT > 1.5 ULN), with bleeding tendency or receiving thrombolytic or anticoagulant therapy;
4. Have clear gastrointestinal bleeding concerns (such as local active ulcer lesions, fecal occult blood + +), a history of gastrointestinal bleeding within 6 months;
5. Symptomatic central nervous system metastasis;
6. Pulmonary hemorrhage ≥ CTCAE level 2 occurred within 4 weeks prior to the first use of the study drug; bleeding occurred at other sites of grade ≥ CTCAE 3 within 4 weeks prior to the first use of the study drug;

***3.2.3 Termination criteria***

1. Medical imaging examination shows that after the disease progresses, the researcher judges that the patient who continues to take the drug cannot benefit;
2. Subjects are still unable to tolerate toxicity after dose adjustment;
3. The subject withdraws the informed consent and requests to withdraw;
4. The subject experienced a pregnancy event during the study;
5. The investigator believes that it is necessary to withdraw from the study.

***3.2.4 Withdraw criteria***

1. Failure to complete at least 1 cycle of clinical trial studies based on non-test drug factors, failure to evaluate safety and efficacy;
2. Serious violation of this study protocol, not according to the prescribed dosage, method and course of treatment.
3. **Study Methods**

**4.1 Drug Administration**

Subjects were randomly divided into two groups: 1) control group, treated with traditional chemotherapy drugs, such as pemetrexed (50 mg/m2, once every 21 days) and docetaxel (75 mg/m2, once every 21 days), alone or in combination with platinum (75 mg/m^2^, once every 21 days); 2) case group, treated with Apapitatin Mesylate (AM) 500 mg/m2 per day for 21 consecutive days) or AM combined with traditional chemotherapy drugs. The patients in both groups were treated for 21 days as a treatment cycle.

The patients were continuously treated by AM for 21 days until PD, intolerable toxicity or patient request to stop; Daily oral dexamethasone 8 mg, 12 h/time before docetaxel, for 3 days. Docetaxel 60~75mg/m2, intravenous infusion for 1 hour, repeated every 3 weeks; or weekly therapy, docetaxel 35～40mg/m2, intravenous infusion for 1 hour, once a week, continuous use 6 weeks, stop for 2 weeks. When AM is combined with TKIs, dose adjustment can be made according to the patient's condition and systemic condition.

**4.2 Dosing cycle**

Patients who achieved complete remission (CR), partial remission (PR), and stable disease (SD) continued to be dosed until disease progression (PD), intolerable toxicity, or the patient requested discontinuation of the drug.

**4.3 Safety Considerations**

The first efficacy evaluation was performed at the end of the second cycle; the efficacy was confirmed at the end of the third cycle, and then the efficacy evaluation was performed every two cycles (this time is based on the calendar days, which is not affected by the withdrawal), and the evaluation time ends at the end of the cycle. Within 7 days (except statutory holidays). The evaluation requires CT or MRI. The imaging techniques used in the same patient evaluation should be the same, and all imaging data should be retained. Subjects with efficacy of CR, PR, and SD were reviewed and confirmed 3 weeks after the first evaluation.

In the case of radiographically confirmed Progressive Disease (PD), the subject discontinued the trial and immediately entered the follow-up period. No other anti-tumor treatments can be performed until PD is present.

For patients who are intolerable due to toxic reaction, if the efficacy evaluation is not performed within 3 weeks before the out of the group, the efficacy evaluation should be performed at the time of grouping, and then the imaging examination should be performed every two cycles at the same frequency until the disease progresses. Or use other anti-tumor treatments; be sure to obtain imaging evidence of PD in such patients.

**4.4 Follow-up**

All subjects should continue to be assessed for safety within 28 days of the end of the last dose. On the 28th day after the end of treatment (±3 days, except for statutory holidays), physical examination, ECOG score, blood routine and blood biochemistry, evaluation of adverse events, concomitant medication and concomitant therapy should be performed.

**4.5** **Adverse events**

Adverse events that have not recovered when apafitini is discontinued should be tracked and final evaluated. Adverse events were followed up to the 28th day after discontinuation.

**4.6 Survival follow-up**

After 28 days of safe follow-up to the end of death or the subject was lost to follow-up or Hengrui termination study, the subject, his family or local physician was asked by phone every 1 month to collect survival (date of death and cause of death) And study the information after the end of treatment (including the treatment received). The follow-up of each survival should be recorded in the follow-up table.

1. **Effectiveness**
   1. **Main endpoints**

**Progression-free survival (PFS)** is the length of time during and after the treatment of a disease, such as cancer, that a patient lives with the disease but it does not get worse.

Definition of evaluable population: All subjects who met the following criteria, ie, received at least 2 treatment cycles (6 weeks) with apatinib and received 1 tumor evaluation after 2 treatment cycles (6 weeks) If the effect is CR, PR or SD, the subject should be reviewed 3 weeks after the first evaluation.

If the subject did not develop disease during the trial, the PFS was defined as the last date until the subject confirmed the last progression free survival. Subjects who discontinued the test (without follow-up imaging) for reasons other than progression of the disease and subjects who received the post-test treatment will be censored as the time to suspend the trial or the time to start the post-test treatment. When the subject is not censored at the time of the discontinuation of the trial or the time of initiation of the post-experimental treatment, the pre-planned sensitivity statistical analysis will further confirm the PFS based only on the time of occurrence of the imaging-confirmed progression. New tumors are not considered to be disease progression events and are not censored as data.

If imaging examinations and evaluations show disease progression, the disease progression date is not the first imaging time to show disease progression, but rather the time of imaging examination to confirm the progression of the disease. If the disease progression is diagnosed by other clinical means, the date of diagnosis will be the date of disease progression.

- 1. **Secondary endpoints**

**Overall survival (OS):** The length of time from either the date of diagnosis or the start of treatment for a disease, such as cancer, that patients diagnosed with the disease are still alive. In a clinical trial, measuring the overall survival is one way to see how well a new treatment works.

**Objective response rate (ORR)** refers to the proportion of patients whose tumors have shrunk to a certain level and maintained for a certain period of time, including cases of CR and PR.

**Disease Control Rate (DCR)** refers to the percentage of confirmed complete remission, partial remission, and stable disease (≥ 8 weeks) in the number of patients with evaluable efficacy.

**Drug safety:** Any adverse events that occurred in all subjects during the clinical study were observed, including abnormalities in clinical symptoms and vital signs, abnormalities in laboratory tests, and recorded clinical manifestations, severity, time, duration, treatment, and prognosis. And determine its correlation with the test drug.

1. **Safety Consideration**
   1. **Adverse event (AE)**

An adverse event means any untoward medical occurrence associated with the use of a drug in humans, whether or not considered drug related. An adverse event (also referred to as an adverse experience) can be any unfavorable and unintended sign, symptom, or disease temporally associated with the use of a drug, without any judgment about causality.

According to regulations, events occurring before and after treatment are also considered to be AEs. Therefore, the safety monitoring AE or SAE report should begin with the subject entering the trial (signing informed consent) until the end of the trial visit.

- 1. **AE classification**

AEs are classified as 0-5 (NCI-CTCAE 4.0) according to NCI's Common Acute and Subacute Toxicity Grading Criteria. AEs not listed in the NCI Toxicity Grading Criteria can be judged according to the following criteria:

I degree (slight): has an uncomfortable feeling, but does not affect normal daily activities;

II degree (moderate): The degree of discomfort is sufficient to reduce or affect normal daily activities;

III degree (severe): a daily activity that cannot work or is normal;

IV degree (fatal): disabling or lethal.

- 1. **Collection of AE**

Details of all AEs occurring during the study must be recorded on the AE form with the following information:

- Description of the event
- Dates of onset and resolution
- Severity
- Action taken
- Outcome
- Relationship to investigational product
- Whether the AE is serious or not

All AEs will be documented in the subject’s source documents (e.g. medical records) and eCRF. Abnormal laboratory test data will be recorded on the CRF form and repeated at least once a week, followed up until normal or at the end of the study. AE occurring within 28 days of the end of the last dose are reported and recorded.

- 1. **Serious Adverse Event**

6.4.1 A ***serious adverse event*** (SAE) or suspected adverse reaction is any untoward medical occurrence that, in the view of either the investigator or sponsor, it:

• Results in death;

• Is considered to be life-threatening;

• Requires inpatient hospitalization or prolongation of existing hospitalization;

• Results in persistent or significant disability, incapacity or substantial disruption of the ability to conduct normal life functions;

• Results in a congenital anomaly or birth defect;

• Is an important medical event that may not result in death, be life-threatening, or require hospitalization but may be considered serious when, based upon appropriate medical judgment, they may jeopardize the patient or subject and may require medical or surgical intervention to prevent one of the outcomes listed in this definition.

***6.4.2 Pregnancy***

Pregnancy during clinical trials should be reported as serious adverse events.

***6.4.3 Disease progression***

Disease progression (including signs and symptoms of progression) should not be reported as a serious adverse event, but death due to disease progression should be reported as a serious adverse event during the trial or safety reporting period. Hospitalization due to symptoms and signs of disease progression should not be reported as a serious adverse event. If the end result of cancer is death during the trial or safety reporting period, the event leading to death must be reported as a serious adverse event.

***6.4.4 Perform other anti-tumor treatments***

If the subject begins other anti-tumor treatments, the non-death adverse event will be reported until the start of a new anti-tumor treatment. If death occurs within the reporting period of a serious adverse event after the end of the study treatment, it must be reported regardless of whether the patient receives other treatment.

***6.4.5 Hospitalization***

Adverse events in clinical studies that result in hospitalization or prolonged hospital stay should be considered serious adverse events. Any initial admission to a medical facility (even if it is shorter than 24 hours) is in compliance with this standard.

Hospitalization does not include the following:

-  Rehabilitation institution
-  Nursing home
-  Conventional emergency room admission
-  On-the-spot surgery (eg outpatient/day/apro-bed surgery)
- Hospitalization or lengthening of hospital stays that are not associated with worsening adverse events are not serious adverse events in themselves, such as:
-  Due to the admission of the original disease, there are no new adverse events, and there is no aggravation of the original disease (such as: in order to check the laboratory abnormalities that persisted until the test);
-  Hospitalization for management reasons (eg annual routine medical examination);
-  hospitalization as specified in the trial protocol during clinical trials (eg, as required by the protocol);
-  elective hospitalization (such as elective cosmetic surgery) that is unrelated to the deterioration of adverse events;
-  The scheduled treatment or surgery should be recorded throughout the trial protocol and/or the individual's individual baseline data;
-  Admitted to hospital for blood use only.

Diagnostic or therapeutic invasive (eg surgery), non-invasive procedures should not be reported as adverse events. However, if the disease status leading to this operation meets the definition of adverse events, it should be reported. If the acute appendicitis that occurs during the adverse event reporting period should be reported as an adverse event, the appendectomy should be recorded as the treatment of the adverse event.

***6.4.6 Overdose***

Overdose refers to the subject taking the test medication within 24 hours (specifically adjusted according to the specific protocol), which is higher than the dose prescribed by the investigator's doctor's advice. All drug overdose, whether related to adverse events/serious adverse events, should be reported as serious adverse events.

***6.4.7 SAE reporting procedure***

Reports of serious adverse events should begin with the signing of the informed consent form from the subject until the last 30 days of the study drug (including the 30th day). During the trial, if serious adverse events occur, they must be reported to the clinical monitor and the main investigator within 24 hours. At the same time, fill out the "New Drug Clinical Research Serious Adverse Event (SAE) Report Form", signed and dated, and faxed. The form is immediately reported to the bidding unit, the leader unit, the ethics committee of the research unit, the State Food and Drug Administration (CFDA), and the food and drug administration of the province (province or city) in the area where the researcher is located.

During the continued drug supply after the study, serious adverse events must be reported to the sponsor within 24 hours. Information on all serious adverse events needs to be recorded in the table of serious adverse events. Serious adverse events that continue during the drug delivery period up to 30 days after the last dose must be reported. Severe adverse events occurring 30 days after the last administration are generally not reported unless suspected to be related to the study drug.

Serious adverse events should be documented in detail, symptoms, time of onset, time of treatment, measures taken, time and manner of follow-up, and outcomes. If the investigator believes that a serious adverse event is not related to the test drug and is potentially related to the study condition (eg, termination of the original treatment, or comorbidity during the trial), then this relationship should be described on the Serious Adverse Events page of the Medical Record Report Form. If the strength of a serious adverse event that is occurring or its relationship to the test drug changes, a follow-up report of the serious adverse event should be sent immediately to the sponsor. All serious adverse events should be followed up to recovery or stabilization.

1. **Data Management**

**7.1Statistical analysis datasets**

Full Analysis Set: Efficacy analysis was performed on all randomized cases and used at least once according to the principle of intention to analysis (ITT). For case data in which the entire course of treatment was not observed, the last observation data was used to carry forward the results to the final test (LOCF).

Per-protocol Set: All cases that meet the test protocol, have good compliance, do not take the drug during the trial period, and complete the case report. No imputation is made for missing data. The efficacy of the drug was simultaneously analyzed statistically for FAS and PPS.

Safety Analysis Set: All patients enrolled in the study, who had used the test at least once, and all patients with post-drug safety records were included in the safety analysis set. This data set is used for security analysis.

1. **Quality Assurance**

Researchers must be clinically trained physicians and work under the direction of a senior professional.

Pre-test clinical wards must meet the requirements of standardization to ensure that the rescue equipment is fully equipped.

It is recommended that the professional nursing staff give the subject medication to learn more about the medication and ensure the compliance of the subject.

The research center must be carried out in strict accordance with the research plan and truthfully entered the case report form.

The auditor should follow the standard operating procedures, supervise the clinical trials, confirm that all data records and reports are correct and complete, all case report forms are entered correctly, and consistent with the original data to ensure that the trials are carried out in accordance with the clinical research protocol.

In the event of SAE, each research unit shall be notified in a timely manner and the study shall be temporarily suspended if necessary.

The research units participating in the trial should accept the audits of the sponsors and the drug regulatory authorities. It is especially important that the researchers and their related personnel should provide convenience and time for the inspection and audit.

1. **Dissemination of Results and Publication Policy**
   1. **Use of Information**

The data may not be used without the permission of Jiangsu Hengrui Pharmaceutical Co., Ltd.

The Scientific Committee has full access to the final data so that appropriate academic analysis and reporting can be conducted on the results of the study.

- 1. **Publish**

All investigators and committee members fully authorize the Scientific Committee to publish the results for the first time or for the first time. No other publications are allowed until the first publication. Any subsequent publication or publication of the study participants (including sub-studies) must be approved by the Scientific Committee and cited for the study and initial publication.

The final decision of any manuscript/summary/newsletter is made by the Scientific Committee after the company has been notified in advance (for internal review and comments). The sponsor may request the name of the sponsor and/or the name of one or more of the sponsors listed or not listed in this publication.

The sponsor may delay the publication or communication for a limited period of time in order to protect the confidentiality or ownership of any information contained therein.

1. **Duration of the Project**

Start time: August 2016, and planned end time: August 2018.

1. **Project Management**

**11.1** **Sponsor Obligations**

The Sponsor monitor or designee will contact and visit the Investigator regularly and will be allowed, on request, to inspect the various records of the trial. The monitor will visit as soon as possible following enrollment of the first subject and at regular intervals during the study as deemed necessary. It will be the monitor’s responsibility to inspect the source documents at regular intervals throughout the study, to verify the adherence to the protocol and the completeness, correctness and accuracy of all eCRF entries. The study monitor will have access to laboratory test reports and any other source records and data needed to verify the entries on the eCRFs, unless restricted by local laws. The Investigator agrees to cooperate with the study monitor to ensure that any problems detected in the course of these monitoring visits are resolved.

This study will be conducted in compliance with Good Clinical Practice, and applicable regulatory requirements.

**11.1 Investigator Obligations**

The investigator is responsible for ensuring that the study is performed in accordance with the protocol, current ICH guidelines on Good Clinical Practice (GCP), and applicable regulatory and country-specific requirements.

1. **Ethics**

**12.1 Ethical Principles**

The study will be conducted in accordance with the principles established by the 18th World Medical Association Joint Conference (Helsinki, 1964) and all subsequent amendments.

**12.2 Laws and regulations**

This study will be conducted in accordance with all laws and regulations.

**12.3 Data Protection**

Patient personal data and researcher personal data that may be included in the database of Jiangsu Hengrui Pharmaceutical Co., Ltd. shall be handled in accordance with all applicable local laws and regulations.

When archiving or processing personal data relating to the investigator and/or patient, Jiangsu Hengrui Pharmaceutical Co., Ltd. shall take all appropriate measures to protect and prevent any unauthorized third party from obtaining such information.

**12.4 Confidentiality of Subject Records**

All materials, materials (verbal or written) and unpublished documents, including the program and CRF, provided to the investigator (or any action taken by the sponsor on behalf of the investigator) are the Jiangsu Hengrui Pharmaceutical Co., Ltd. Exclusive property.

The investigator or any member of his/her team shall not disclose such materials or materials to unauthorized persons without the prior written consent of Jiangsu Hengrui Pharmaceutical Co., Ltd.

In addition to the information permitted by the regulations, the researcher should keep all information received, obtained or derived during the course of the study confidential and take all necessary steps to ensure that it is not compromised.

**12.5 Record Retention**

The investigator should arrange for the storage of the research documents until the end of the study. In addition, for patient record keeping, researchers should follow specific local regulations/guidance.

Unless otherwise stated in the investigator's agreement, in accordance with other standards and/or local laws, the investigator is advised to keep the research document for at least five years after the study is completed or interrupted.

**12.6 Study early interruption**

Jiangsu Hengrui Pharmaceutical Co., Ltd. may terminate the study at any time and for any reason; the decision to discontinue the study will be communicated to the participating researchers in writing.

Similarly, if the researcher decides to withdraw from the study, he/she must notify the company in writing.

If applicable, the Ethics Committee (IRB) and the health supervisory authority should be notified in accordance with local regulations.

**12.7** **Audit and Inspection**

The investigator agrees to have the sponsor's inspectors/regulators' inspectors directly review the subject's research records for review and understand that these individuals are bound by the principle of occupational confidentiality and therefore should not disclose any personal identity or personal medical care of the patient’s information.

The investigator will make every effort to assist in the conduct of inspections and inspections, so that the inspectors/inspectors have access to all necessary equipment, data, and documentation.

The confidentiality of the verification data and the protection of the patient should be respected during these inspections.

The researcher should immediately communicate the results and information given by the regulatory agency to the sponsor.

The investigator shall take appropriate measures at the request of the sponsor to take corrective action on all issues identified during the inspection or inspection process.

1. **Informed Consent Forms**

Prior to participation, the study procedures and any known or likely risks will be explained to the subjects by the investigator or other medically qualified co-investigator. An Informed Consent Form will also be provided containing all the required information. Any questions will be answered and the patient will then be given sufficient time to consider their participation in the study before signing a consent form. Subjects should receive a copy of the Informed Consent Form.

The Investigator will explain that the subjects are completely free to refuse to enter the study or to withdraw from it at any time, without any consequences for their further care and without the need to justify.

Each subject will be informed that the subject’s source medical records may be checked by representatives from the Sponsor or from a regulatory agency, in accordance with applicable regulations. However, they should be made aware that all information will be treated with confidentiality, and a study ID code or number will identify them.

1. **Financing and Insurance**

**14.1 Financial Disclosure**

The investigator is responsible for updating the Sponsor if there are any changes that would affect their Financial Disclosure during the conduct of the study.

**14.2** **Liability and Insurance Conditions**

In case of any damage or injury occurring to a subject in association with the trial medication or participation in the study, Jiangsu Hengrui Pharmaceutical Co., Ltd. has insurance covers. A copy of this policy is on file at Jiangsu Hengrui Pharmaceutical Co., Ltd.
