# Supplementary material for: Apatinib Mesylate in the treatment of advanced progressed lung adenocarcinoma patients with EGFR-TKI resistance —A Multicenter Randomized Trial
Source: Sci Rep. 2019 Sep 30;9:14013. doi: 10.1038/s41598-019-50350-6 (PMC6768876; doi:10.1038/s41598-019-50350-6)
Supplement: Supplementary file 4 — Supplementary Materials 1 [file 41598_2019_50350_MOESM4_ESM.pdf]

Apatinib Mesylate in the treatment of advanced progressed lung adenocarcinoma patients with EGFR-TKI resistance

—A Multicenter Randomized Trial

Ping Fang[1*], Liqin Zhang[2], Xianru Zhang[3], Jiawen Yu[4], Jun Sun[5], Qi-an Jiang[6], Mingbao Zha[7], Anastasia P. Nesterova[8], Hongbao Cao[8,9*]

[1]Department of Respiratory, the People's Hospital of Tongling, Tongling, Anhui province, 244000, China;

[2]Department of Respiratory, Yijishan Hospital of Wannan Medical College, No. 2 Zheshan West Road, Wuhu, Anhui Province, 241000, China;

[3]Department of Respiratory, Tongling Municipal Hospital, No. 2999 Changjiang West Road, Tongling, Anhui Province, 244099, China;

[4]Department of Respiratory, Anqing First People's Hospital, No. 42 Xiaosu Road, Anqing, Anhui Province, 246000, China;

[5]Department of Respiratory, Xuancheng People's Hospital, No. 15 Huancheng North Road, Xuancheng, Anhui Province, 242000, China;

[6]Department of Respiratory, Anqing Municipal Hospital, No.172 Renmin Road, Yingjiang District, Anqing, Anhui Province, 246000, China;

[7]Department of Respiratory, Wuhu City Hospital of Traditional Chinese Medicine, No. 240 Jiuhua Middle Road, Jinghu District, Wuhu, Anhui Province, 2461002, China;

[8]Department of Biology Solution, Elsevier, 1150 18th St NW, Washington, DC 20036, USA.

[9]Department of Psychiatry, First Hospital/First Clinical Medical College of Shanxi Medical University, Taiyuan, Shanxi Province, 030001, China.

```
  ___  ____  ____  ____  ____(R)
 /__    /   ____/   /   ____/
___/   /   /___/   /   /___/
  Statistics/Data Analysis
```

User: KM(lifetable)/log-rank test
Project: 20190809

1 . ltable OS die, survival by(group)

Interval		Beg. Total	Deaths	Lost	Survival	Std. Error	[95% Conf.	Int.]
**group = 0**								
30	31	29	3	0	0.8966	0.0566	0.7126	0.9654
35	36	26	1	0	0.8621	0.0640	0.6731	0.9459
60	61	25	3	0	0.7586	0.0795	0.5594	0.8769
84	85	22	0	1	0.7586	0.0795	0.5594	0.8769
90	91	21	1	0	0.7225	0.0835	0.5208	0.8505
105	106	20	1	1	0.6854	0.0870	0.4819	0.8225
120	121	18	3	0	0.5712	0.0943	0.3687	0.7303
135	136	15	0	1	0.5712	0.0943	0.3687	0.7303
150	151	14	1	0	0.5304	0.0960	0.3298	0.6959
180	181	13	3	0	0.4080	0.0964	0.2225	0.5858
270	271	10	1	0	0.3672	0.0950	0.1898	0.5466
315	316	9	1	0	0.3264	0.0928	0.1586	0.5063
330	331	8	1	0	0.2856	0.0897	0.1290	0.4645
420	421	7	1	1	0.2417	0.0860	0.0982	0.4193
450	451	5	0	1	0.2417	0.0860	0.0982	0.4193
690	691	4	1	0	0.1812	0.0831	0.0556	0.3644
720	721	3	0	1	0.1812	0.0831	0.0556	0.3644
885	886	2	0	1	0.1812	0.0831	0.0556	0.3644
1395	1396	1	0	1	0.1812	0.0831	0.0556	0.3644
**group = 1**								
14	15	37	0	1	1.0000	0.0000	.	.
25	26	36	1	0	0.9722	0.0274	0.8187	0.9960
29	30	35	1	0	0.9444	0.0382	0.7957	0.9858
42	43	34	1	0	0.9167	0.0461	0.7635	0.9723
57	58	33	1	0	0.8889	0.0524	0.7305	0.9568
58	59	32	0	1	0.8889	0.0524	0.7305	0.9568
73	74	31	1	0	0.8602	0.0580	0.6961	0.9393
84	85	30	1	0	0.8315	0.0628	0.6628	0.9206
85	86	29	1	0	0.8029	0.0668	0.6303	0.9008
92	93	28	0	1	0.8029	0.0668	0.6303	0.9008
97	98	27	0	1	0.8029	0.0668	0.6303	0.9008
100	101	26	0	1	0.8029	0.0668	0.6303	0.9008
103	104	25	1	1	0.7701	0.0717	0.5914	0.8782
134	135	23	0	1	0.7701	0.0717	0.5914	0.8782
135	136	22	1	1	0.7343	0.0768	0.5490	0.8529
139	140	20	0	1	0.7343	0.0768	0.5490	0.8529
143	144	19	1	0	0.6956	0.0819	0.5039	0.8251
159	160	18	1	0	0.6570	0.0860	0.4614	0.7960
165	166	17	1	0	0.6183	0.0892	0.4208	0.7657
181	182	16	1	0	0.5797	0.0916	0.3820	0.7342
182	183	15	1	0	0.5410	0.0933	0.3448	0.7016
196	197	14	1	0	0.5024	0.0943	0.3089	0.6681
197	198	13	1	0	0.4638	0.0946	0.2744	0.6334
234	235	12	1	0	0.4251	0.0943	0.2412	0.5978
242	243	11	1	0	0.3865	0.0933	0.2093	0.5611
269	270	10	1	0	0.3478	0.0916	0.1787	0.5233
279	280	9	1	0	0.3092	0.0892	0.1495	0.4843
286	287	8	1	0	0.2705	0.0860	0.1217	0.4442
289	290	7	2	0	0.1932	0.0769	0.0712	0.3595
291	292	5	1	0	0.1546	0.0706	0.0491	0.3147
299	300	4	1	0	0.1159	0.0626	0.0296	0.2676
357	358	3	1	0	0.0773	0.0523	0.0136	0.2177
401	402	2	0	1	0.0773	0.0523	0.0136	0.2177
485	486	1	0	1	0.0773	0.0523	0.0136	0.2177

```
2 . stset PFS , failure( progress ==1)

     failure event:  progress == 1
obs. time interval:  (0, PFS]
 exit on or before:  failure

------------------------------------------------------------------------------
        104  total observations
         38  event time missing (PFS>=.)                          PROBABLE ERROR
------------------------------------------------------------------------------
         66  observations remaining, representing
         46  failures in single-record/single-failure data
      8,466  total analysis time at risk and under observation
                                                at risk from t =         0
                                     earliest observed entry t =         0
                                          last observed exit t =       465

3 . sts test group

         failure _d:  progress == 1
   analysis time _t:  PFS
```

Log-rank test for equality of survivor functions

group	Events observed	Events expected
0	25	19.20
1	21	26.80
Total	46	46.00

```
            chi2(1) =       3.25
            Pr>chi2 =     0.0714

4 . stset OS , failure( die ==1)

     failure event:  die == 1
obs. time interval:  (0, OS]
 exit on or before:  failure

------------------------------------------------------------------------------
        104  total observations
         38  event time missing (OS>=.)                           PROBABLE ERROR
------------------------------------------------------------------------------
         66  observations remaining, representing
         47  failures in single-record/single-failure data
     14,218  total analysis time at risk and under observation
                                                at risk from t =         0
                                     earliest observed entry t =         0
                                          last observed exit t =     1,395

5 . sts test group

         failure _d:  die == 1
   analysis time _t:  OS
```

Log-rank test for equality of survivor functions

group	Events observed	Events expected
0	21	23.42
1	26	23.58
Total	47	47.00

```
            chi2(1) =       0.54
            Pr>chi2 =     0.4617
```
