# Supplementary material for: Apatinib Mesylate in the treatment of advanced progressed lung adenocarcinoma patients with EGFR-TKI resistance —A Multicenter Randomized Trial
Source: Sci Rep. 2019 Sep 30;9:14013. doi: 10.1038/s41598-019-50350-6 (PMC6768876; doi:10.1038/s41598-019-50350-6)
Supplement: Supplementary file 5 — Supplementary Materials 2 [file 41598_2019_50350_MOESM5_ESM.pdf]

Apatinib Mesylate in the treatment of advanced progressed lung adenocarcinoma patients with EGFR-TKI resistance —A Multicenter Randomized Trial

Ping Fang[1*], Liqin Zhang[2], Xianru Zhang[3], Jiawen Yu[4], Jun Sun[5], Qi-an Jiang[6], Mingbao Zha[7], Anastasia P. Nesterova[8], Hongbao Cao[8,9*]

[1]Department of Respiratory, the People's Hospital of Tongling, Tongling, Anhui province, 244000, China;
[2]Department of Respiratory, Yijishan Hospital of Wannan Medical College, No. 2 Zheshan West Road, Wuhu, Anhui Province, 241000, China;
[3]Department of Respiratory, Tongling Municipal Hospital, No. 2999 Changjiang West Road, Tongling, Anhui Province, 244099, China;
[4]Department of Respiratory, Anqing First People's Hospital, No. 42 Xiaosu Road, Anqing, Anhui Province, 246000, China;
[5]Department of Respiratory, Xuancheng People's Hospital, No. 15 Huancheng North Road, Xuancheng, Anhui Province, 242000, China;
[6]Department of Respiratory, Anqing Municipal Hospital, No.172 Renmin Road, Yingjiang District, Anqing, Anhui Province, 246000, China;
[7]Department of Respiratory, Wuhu City Hospital of Traditional Chinese Medicine, No. 240 Jiuhua Middle Road, Jinghu District, Wuhu, Anhui Province, 2461002, China;
[8]Department of Biology Solution, Elsevier, 1150 18th St NW, Washington, DC 20036, USA.
[9]Department of Psychiatry, First Hospital/First Clinical Medical College of Shanxi Medical University, Taiyuan, Shanxi Province, 030001, China.

Pathways to understand the treatment effect of Apatinib on lung adenocarcinoma

To understand the role of Apatinib Mesylate in the treatment of lung adenocarcinoma, we analyzed a literature-based database of biomolecular interactions ("ResNet – 2018", Pathway Studio, Elsevier Inc.) which covers more than 50 million references – records from the published papers[10]. The purpose of analysis was to identify all possible mechanisms of action of Apatinib Mesylate and find cellular signaling pathways linking Apatinib Mesylate to lung adenocarcinoma. In ResNet - 2018 database Apatinib Mesylate (rivoceranib, YN 968D1) has connections with 248 biologically related terms (diseases, adverse effects, proteins, cell, cellular processes, treatments, compounds), therefore at least 248 individual facts about the Apatinib Mesylate have been published. For example, Apatinib Mesylate was mentioned along with more 100 diseases, where gastric cancer and non-small cell lung cancer have been cited most frequently. In addition, 6 clinical trials related to usage of Apatinib Mesylate in different types of lung cancer were published over last 5 years and cited in the database (please refer to Apatinib_LA, online available at http://gousinfo.com/database/Data_Genetic/Apatinib_LA.xlsx). Apatinib is connected to many cellular processes, clinical parameters and cell types in the database (Figure 3, Table 4, and supplemental materials: Apatinib_LA→Ref4Fig2).

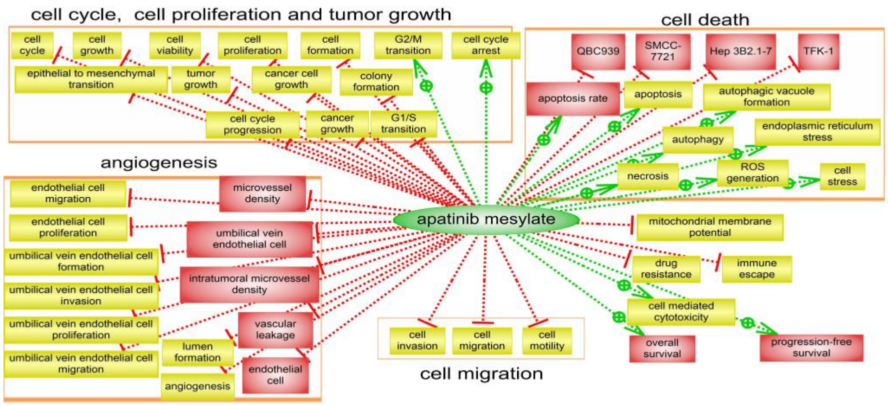

Figure 1. Functional effects of Apatinib Mesylate on tumor. There are 4 main classes of biological cellular processes in which Apatinib plays the key role according to Elsevier Pathway Studio. It inhibits cell cycle and tumor cells proliferation, inhibits cell migration (metastasis), induces cancer cell death. And most importantly, Apatinib inhibits angiogenesis and progressing of tumor vascularization.

Table 1. Publications about the role of Apatinib Mesylate in oncology-related diseases.

Therapeutic Area	# of References	Type of Extracted Facts	Extracted Relationship	First three MedLine Ref ID
Gastrointestinal Diseases	30	ClinicalTrial	rivoceranib ---> gastric cancer	102510469, 102509806, 102697838
Pulmonology	18	ClinicalTrial	rivoceranib ---> non-small cell lung cancer	103083041, 103050411, 103129256
Hepatology	13	ClinicalTrial	rivoceranib ---> hepatocellular carcinoma	103046979, 103398122, 102772029
general oncology	12	ClinicalTrial	rivoceranib ---> cancer	102544737, 101497704, 102863367
Hematologic Diseases	10	Regulative role (inhibitor)	rivoceranib ---| leukemia	29490645:13, 22212563:4, 22212563:3
…				
Pulmonology Diseases	9	ClinicalTrial	rivoceranib ---> small cell lung cancer	102980809, 102945852, 103389087
Pulmonology Diseases	5	ClinicalTrial	rivoceranib ---> lung cancer	102704767, 103411967, 103135977
Pulmonology Diseases	3	ClinicalTrial	rivoceranib ---> lung adenocarcinoma	103376737, 102493582, 102691871

<table>
<tr><td>Pulmonology Diseases</td><td>3</td><td>Regulative role (inhibitor)</td><td>rivoceranib ---|
lung squamous cell carcinoma</td><td>30547193:13,
30038504:5,
30547193:100</td></tr>
<tr><td>Pulmonology Diseases</td><td>1</td><td>Regulative role (inhibitor)</td><td>rivoceranib ---|
alveolar soft part sarcoma</td><td>28679123:8</td></tr>
<tr><td>Pulmonology Diseases</td><td>1</td><td>Regulative role (inhibitor)</td><td>rivoceranib ---| epithelioid hemangioendothelioma</td><td>29137048:12</td></tr>
<tr><td>Pulmonology Diseases</td><td>1</td><td>ClinicalTrial</td><td>rivoceranib --->
lung disease</td><td>103389256</td></tr>
<tr><td>Pulmonology Diseases</td><td>1</td><td>ClinicalTrial</td><td>rivoceranib --->
lung metastasis</td><td>102702323</td></tr>
<tr><td>Pulmonology Diseases</td><td>1</td><td>ClinicalTrial</td><td>rivoceranib --->
lung neoplasm</td><td>102852798</td></tr>
</table>

Apatinib has two dozen of molecular targets in the ResNet -2018 database. The drug influences at least 25 proteins which were shown to have connections to lung adenocarcinoma (Table 5, Figure 4, and supplemental materials: Apatinib_LA→Ref4Fig3). In his original work Tian and co-authors reported that Apatinib inhibits the function of VGFR2 (VEGF-A receptor-2 or kinase insert domain receptor, KDR) with high selectivity in human tumor cell lines through binding to the receptor and blocking its VEGF-stimulated phosphorylation. Tian showed that Apatinib also inhibits function of several other tyrosine kinase receptors RET, KIT, SRC and PDGFRB[11]. The inhibitory effects of Apatinib on molecular cascades related to vascular endothelial growth factors were confirmed in several studies. However, the nature of the inhibition is still under investigation. Wen showed that in liver cancer cell line Apatinib inhibits both VGFA and VGFR2 indirectly through regulation of their level of the expression[12].

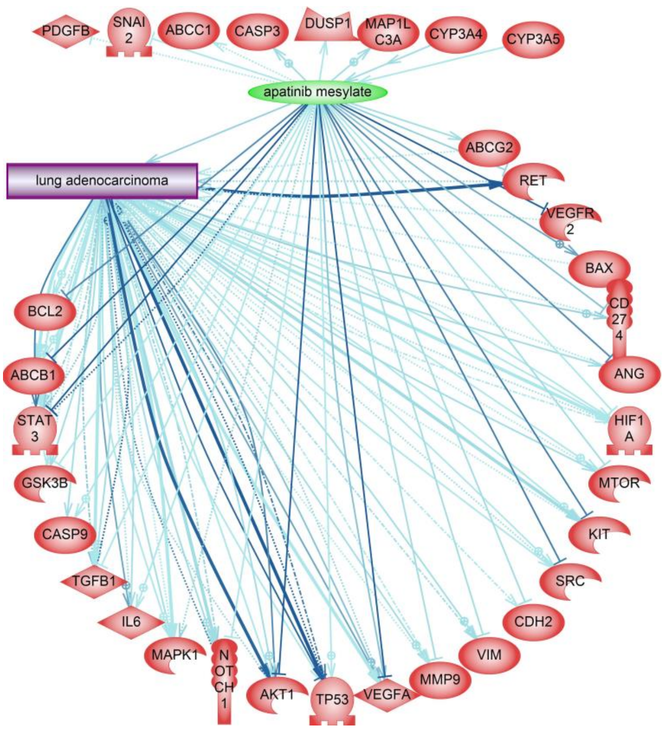

Figure 2. Protein targets of Apatinib Mesylate.

Table 2. Protein targets of Apatinib

Relation	Type of association	# of citations	PMID	Last pub Year	Title
rivoceranib --+> IL6	Indirect regulation of expression	1	29886786	2018	Apatinib, a novel tyrosine kinase inhibitor, suppresses tumor growth in cervical cancer and synergizes with Paclitaxel
rivoceranib ---> ABCC1	Indirect unknown regulation	1	26020064	2015	Apatinib: A promising oral antiangiogenic agent in the treatment of multiple solid tumors
rivoceranib --+> TP53	Indirect regulation of expression	1	29972411	2018	Pioglitazone Induces Cardiomyocyte Apoptosis and Inhibits Cardiomyocyte Hypertrophy Via VEGFR-2 Signaling Pathway.
rivoceranib ---> VEGFA	Indirect regulation of expression	4	30686547	2019	Apatinib regulates the cell proliferation and apoptosis of liver cancer by regulation of VEGFR2/STAT3 signaling.
rivoceranib ---| KIT	Direct Regulation	7	30863099	2019	Treatment of adult brainstem glioma with combined antiangiogenic therapy: a case report and literature review.

Using ResNet-2018 and Pathways Studio application we build the molecular model (pathway) of inhibition of VEGFR2 signaling cascade by Apatinib in general cancer cell (Figure 5 and supplemental materials: Apatinib_LA→Ref4Fig4). The pathway model about the role of Apatinib in cross-talk between VEGFR2 and EGFR signaling in lung adenocarcinoma was also build (Figure 6 and supplemental materials: Apatinib_LA→Ref4Fig5), which indicated that Apatinib may block parts of EGFR or VGFR2-related intracellular cascades through the decrease in the activated status of proteins that were relevant to cell migration and proliferation, inhibit ABCB1 and ABCG2 to prolong the treatment effect of EGFR blocking, and decrease BCL2 activity resulting in more pronounced apoptosis of cancer cell.

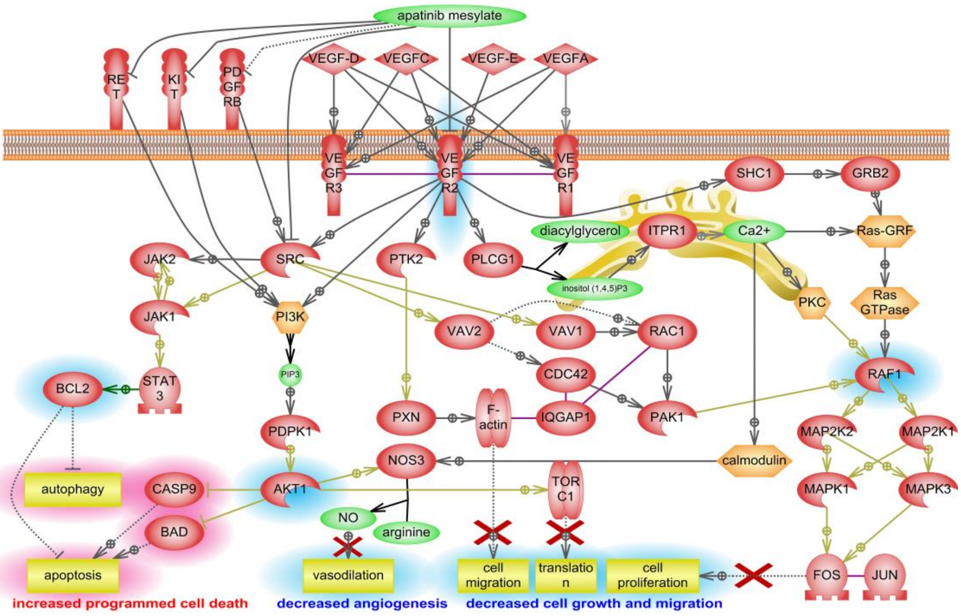

Figure 3 Apatinib Mesylate Targets in VEGFR2 signaling pathway. Soluble VEGFs binds to VGFR2 on the cell membrane inducing the receptor's dimerization with following autophosphorylation and signal transduction. The autophosphorylation of tyrosine residues within VEGFR2 creates docking sites for several intracellular mediators such as modular SRC, SCH1 and phospholipase C (PLCG1). Further cascades are highly intercrossed. Only major protein-protein connections are shown for simplicity.

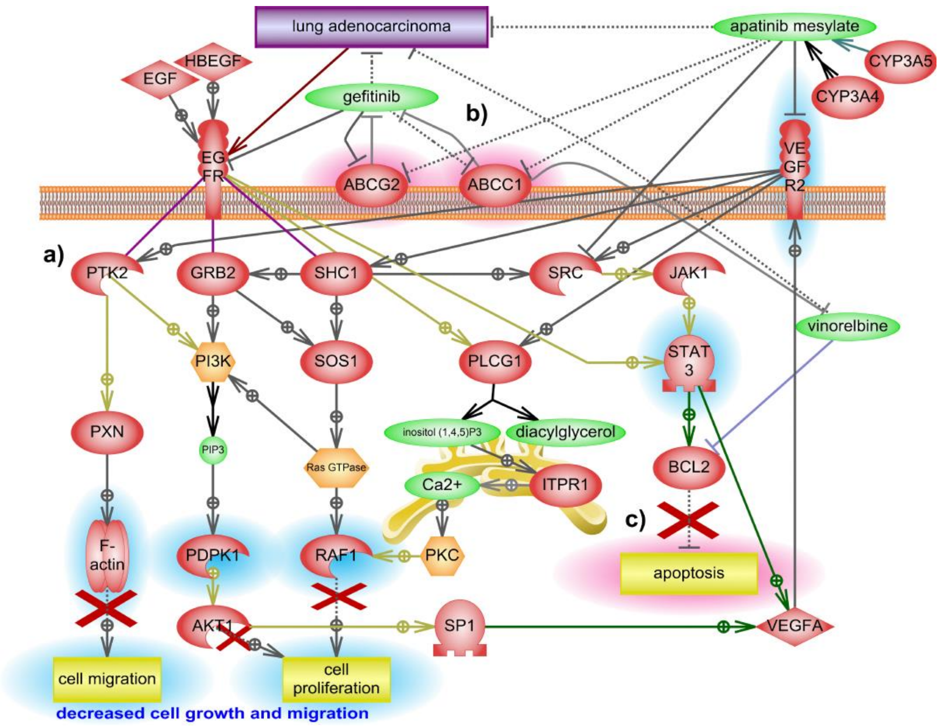

Figure 4. Mechanism of action of Apatinib Mesylate in lung adenocarcinoma. EGFR signaling is the major trigger for vascular or tumor cell proliferation in LA. Apatinib may block parts of EGFR -related intracellular cascades: a) through the decrease in the activated status of proteins that are stimulated by both EGFR and VGFR2 receptors. That leads to decrease of cancer cell migration and proliferation; b) through inhibiting of ABCB1 and ABCG2 resulting in the more long effect of used drugs on blocking of the EGFR; c) by decrease BCL2 activity resulting in more pronounced apoptosis of cancer cell.
